# The PAX6-ZEB2 axis promotes metastasis and cisplatin resistance in non-small cell lung cancer through PI3K/AKT signaling

**DOI:** 10.1038/s41419-019-1591-4

**Published:** 2019-04-25

**Authors:** Dong-ming Wu, Ting Zhang, Ya-bin Liu, Shi-hua Deng, Rong Han, Teng Liu, Jing Li, Ying Xu

**Affiliations:** Clinical Laboratory, The First Affiliated Hospital of Chengdu Medical College, Chengdu, Sichuan 610500 China

**Keywords:** Non-small-cell lung cancer, Metastasis, Tumour biomarkers

## Abstract

Paired-box 6 (PAX6) is an important transcription factor required for the function of human neuroectodermal epithelial tissues. Previous studies have suggested that it is also expressed in several types of tumors and has an oncogenic role. However, little is known about its role in non-small cell lung cancer (NSCLC). Here, we found that PAX6 expression levels were upregulated in human lung cancer tissues and correlated with poor clinical outcomes. PAX6 overexpression significantly promoted NSCLC epithelial-to-mesenchymal transition (EMT) and metastasis, whereas its knockdown inhibited these processes. PAX6 is commonly correlated with EMT-mediated stem cell transformation, thereby inducing cisplatin resistance. Using the RT^2^ Profiler PCR Array, we found that *WNT5A*, *EGFR*, and *ZEB2* were differentially regulated in response to PAX6 modulation. In addition, PAX6 directly bound to the promoter region of *ZEB2*. ZEB2 knockdown significantly reduced the expression and function of PAX6. ZEB2 was upregulated upon PAX6 overexpression and downregulated upon PAX6 knockdown, whereas E-cadherin expression negatively correlated with PAX6 levels. Moreover, p-PI3K and p-AKT were significantly enhanced by PAX6, which was reversed by the addition of the PI3K-AKT inhibitor, LY294002. These data suggest that PAX6 can mediate E-cadherin downregulation through the PI3K/AKT signaling pathway by directly binding the promoter region of *ZEB2*, thereby mediating cell migration, stem cell transformation, and cisplatin resistance; and ultimately, affecting survival in NSCLC patients.

## Introduction

Lung cancer is the leading cause of cancer-related deaths in both men and women worldwide.^1^ Non-small cell lung cancers (NSCLCs) account for 80% of lung cancers, and of these, ~50% are adenocarcinomas.^2^ Despite advances in the treatment of lung cancer, distant metastasis remains the main factor contributing to disease-associated death.^3^ The current first-line treatment for the majority of metastatic NSCLCs remains limited to platinum-based chemotherapy, which is frequently accompanied by rapid development of drug resistance.^4^ Accordingly, elucidating the molecular mechanisms underlying the development of NSCLC and discovering therapeutic targets to overcome chemoresistance are crucial for establishing effective treatments for NSCLC.

Epithelial-to-mesenchymal transition (EMT) is characterized by the loss of epithelial differentiation and the acquisition of a mesenchymal phenotype.^3^ Moreover, during EMT, cancer cells lose their polarity and adhesion, and gain migratory and invasive capabilities that are typical of mesenchymal cells; it is also a potential cellular program through which tumor cells gain metastatic features.^5^ Although this process was initially recognized to be important during embryogenesis,^5–7^ it is now known to occur in cancer stem cells, drug resistance, and immunosuppression during cancer progression.^8–10^ Moreover, numerous studies have shown that signaling pathways such as PI3K/AKT, MAPK, TGF-β, NF-κΒ, Notch, and Wnt, among others, play crucial roles in EMT-related invasion and metastasis.^11,12^

Paired-box 6 (PAX6), a PAX family member that is encoded by a gene located on chromosome 11p13 in humans, plays an important role in the development of human eyes and in embryogenesis.^13,14^ In addition, PAX6 is an important transcription factor for the function of the central nervous system, stem cells, and the pancreas, as well as for neuroectodermal cell differentiation and apoptosis.^14–17^ However, its expression in tumors, suggesting an oncogenic role, was only recently discovered. Some studies reported that PAX6 is frequently expressed in retinoblastoma, pancreatic tumors, and intestinal tumors^18–20^ and is highly expressed in pancreatic, brain, and breast cancer cell lines.^21,22^ Moreover, it has been reported that PAX8 and PAX5 are highly expressed in lung cancer.^23^ However, little is known about the expression and function of PAX6 in NSCLC.

In this study, we explored the role of PAX6 in NSCLC. We determined that PAX6 is highly expressed in NSCLC and is significantly correlated with reduced overall survival (OS) duration. In addition, this protein was found to promote cell migration and invasion and to contribute to EMT in NSCLC. We also found that PAX6 is correlated with EMT-mediated stem cell transformation, thereby inducing cisplatin resistance. Moreover, it was found to regulate the transcriptional activity of zinc finger E-box-binding protein 2 (ZEB2) and to bind directly to the promoter region of *ZEB2*. Further, the PAX6-ZEB2 axis was determined to promote metastasis by mediating E-cadherin downregulation through the PI3K/AKT pathway in NSCLC. Collectively, our findings suggest that PAX6 might be a novel therapeutic target to overcome metastasis and cisplatin resistance in NSCLC.

## Materials and methods

### Reagents

Antibodies against PAX6 (12323-1-AP), E-cadherin (20874-1-AP), N-cadherin (22018-1-AP), vimentin (10366-1-AP), FSP-1 (16105-1-AP), GAPDH (66004-1-Ig), CD44 (15675-1-AP), CD133 (18470-1-AP), and ALCAM (21972-1-AP), as well as secondary antibodies were obtained from Proteintech Group, Inc. (Wuhan, China); antibodies against EGFR (201012-3F12), WNT5A (619919), ZEB2 (505705), Ki67 (200296), AKT (200323-5A11), phospho-AKT (Ser473) (310021), PI3K (220742), and phospho-PI3K p85alpha (Tyr607) (340790) were purchased from Zenbio (Chengdu, China). The PI3K-AKT inhibitor LY294002 was purchased from the Beyotime Institute of Biotechnology (Shanghai, China). Tissue arrays were purchased from Outdo Biotech Co., Ltd. (Shanghai, China). All other kits and reagents were purchased from the Beyotime Institute of Biotechnology (Shanghai, China).

### Cell culture

The NSCLC cell lines A549 and SPC-A-1 and the normal lung epithelial cell line BEAS-2B were maintained in RPMI-1640 medium supplemented with 10% fetal bovine serum (FBS), 10 mM l-glutamine, and 5 mg/mL penicillin/streptomycin at 37 °C with 5% CO_2_. All media and supplements were purchased from Invitrogen (Carlsbad, CA, USA).

### PAX6 knockdown and overexpression

PAX6 was silenced in A549 cells with siRNA (RiboBio Co., Ltd., Guangzhou, China), according to the manufacturer’s instructions; the target sequences were as follows: si-h-PAX6_001, GCGACTCCAGAAGTTGTAA; si-h-PAX6_002, GCAGACGGCATGTATGATA; si-h-PAX6_003, GCTTCACCATGGCAAATAA. The corresponding negative control was purchased from RiboBio Co., Ltd. To stably knock down PAX6 in cells, siRNA targeting the si-h-PAX6_002 coding sequence 5′-GCAGACGGCATGTATGATA-3′ was designed and inserted into a pGMLV-SC5RNAi lentiviral vector (Genomeditech Co., Ltd, Shanghai, China); a scramble siRNA was used as a negative control.

To overexpress PAX6 in cells, an expression construct was generated by subcloning PCR-amplified, full-length human *PAX6* cDNA into a pGMLV-CMV-PAX6 lentiviral vector (Genomeditech); an empty vector was used as the negative control. These procedures were performed, as described previously.^24^ The knockdown and overexpression efficiencies were evaluated by quantitative reverse transcription PCR (RT-qPCR) and western blotting.

### ZEB2 knockdown

ZEB2 was silenced in A549 cells with siRNA (RiboBio Co., Ltd., Guangzhou, China), according to the manufacturer’s instructions; the target sequences were as follows: si-h-ZEB2_001, GGAGTTACTTCTCCTAATA; si-h-ZEB2_002, GAAGCTACGTACTTTAATA; si-h-ZEB2_003, GCACTAGTCCCTTTATGAA. The corresponding negative control was purchased from RiboBio Co., Ltd. The knockdown efficiency was evaluated by RT-qPCR and western blotting.

### Total RNA extraction and RT-qPCR

Total RNA was extracted from three cell lines (A549, SPC-A-1, BEAS-2B) using a total RNA extraction kit (Solarbio, Beijing, China), according to the manufacturer’s instructions. RNA concentrations were determined using a NanoDrop 2000 spectrophotometer (Thermo Scientific, Waltham, MA, USA). Around 1 µg of total RNA was reversed transcribed using an iScript cDNA synthesis kit (Bio-Rad, Hercules, CA, USA) to synthesize cDNA. qPCR was performed using a CFX96 Real-time System (Bio-Rad) with SYBR Green Supermix (Bio-Rad). Both procedures were performed in accordance with the manufacturer’s instructions. The sequences of the primers used in this study are listed in Additional file 1, Table S1.

### Western blotting

Protein samples were resolved by sodium dodecyl sulfate polyacrylamide gel electrophoresis on 12% gels and transferred to nitrocellulose membranes, which were then blocked for 1 h at room temperature in Tris-buffered saline containing 0.1% Tween-20 and 5% fat-free milk. Primary antibody incubation was performed for 18 h at 4 °C. Then, membranes were stained at room temperature for 1 h with secondary antibodies conjugated to horseradish peroxidase, and visualized with enhanced chemiluminescence (SuperSignal; Pierce, Rockford, IL) or ECL Plus (Amersham Pharmacia Biotech, Buckinghamshire, UK) substrates according to the manufacturers’ instructions.

### Cell invasion and wound healing assays

Transwell migration assays (without Matrigel) and Matrigel invasion assays were performed, as previously described.^25^ For wound healing assays, cells were serum-starved for 24 h for cell cycle synchronization, and a confluent cell monolayer (seeded in 6-well plates) was scratched with sterile 200-μL pipette tips to artificially create wounds. The wound healing process was observed and photographed at a magnification of 100×, at the indicated time points.

### Immunofluorescence (IF)

Cultured cells were fixed with 4% paraformaldehyde, washed twice with PBS, and blocked with PBS containing 10% normal goat serum. Then, the samples were stained with E-cadherin, N-cadherin, vimentin, FSP-1, CD44, CD133, or ALCAM polyclonal antibodies overnight at 4 °C, washed twice with PBS, stained with Cy3 (red)-conjugated secondary antibody for 2 h at 37 °C, and washed twice before imaging. All IF images were obtained with an Olympus BX51 microscope equipped with a 20× or 40× objective lens (Olympus, Tokyo, Japan) and a DP50 camera (Olympus). Images were processed using DPC controller software (Olympus).

### Cell viability assays

Cell viability was assessed by colony formation and cell counting kit-8 (CCK-8) assays. Briefly, cells were plated at 500 cells per well in a 6-well plate (Corning, Corning, NY, USA) after being treated with different concentrations of cisplatin (0, 0.25, 0.5, 1 μg/mL). Cells were cultured for 10 days with medium changes every 3 days. Colonies were washed with PBS, fixed in methanol, and stained with crystal violet. The CCK-8 assay was performed according to the manufacturer’s instructions.

### Flow cytometry

Apoptosis was measured by flow cytometry using an Annexin V-PE/7-AAD apoptosis detection kit (KeyGEN, Jiangsu, China), according to the manufacturer’s instructions. A549 cells treated without or with cisplatin at 1 μg/mL were digested with trypsin without EDTA. The cells were harvested and washed with PBS. Tumor cells were stained with 7-AAD for 15 min. After the reaction, 450 μL of Binding Buffer was added, then 1 μL of Annexin V-PE was added at room temperature in the dark, and the mixture was incubated for 15 min. The cells were analyzed using a flow cytometer (FACSCalibur, Becton-Dickinson, USA).

### Sphere formation assay

The A549 cells in good growth state were digested, centrifuged and washed twice with sterile PBS after removing the serum-containing medium. The cells were then resuspended in Dulbecco’s modified Eagle medium/F12 medium containing 20 ng/mL epidermal growth factor, 20 ng/mL basic fibroblast growth factor and 1 × B27 supplement. Cells were cultured in six-well ultra-low-attachment plates at a density of 5000 cells/well and incubated at 37 °C with 5% CO_2_ for 7–10 days. Images of representative tumor-spheres were taken and quantified under microscopy.

### Immunohistochemistry (IHC)

Tissue arrays were dewaxed, and antigens were retrieved using high pressure. Endogenous peroxidases were blocked with 3% hydrogen peroxide for 10 min. After the addition of normal goat serum for 30 min, the tissues were incubated with the primary antibody at 4 °C overnight, washed with phosphate-buffered saline (PBS), and then incubated with a biotin-conjugated secondary antibody (ZSGB-BIO, Beijing, China) for 30 min at 37 °C. After washing, the sections were incubated with horseradish peroxidase complex for 30 min at 37 °C and visualized using diaminobenzidine (DAB). All immunohistochemical images were obtained using an Olympus BX51 microscope equipped with a 20×, 40×, or 100× objective lens (Olympus) and a DP 50 camera (Olympus). Images were processed using DPC controller software (Olympus).

PAX6 expression was assessed by multiplying scores representing the percentage and intensity of staining. Staining intensity was graded as 0 (no staining), 1 (weak staining=light yellow), 2 (moderate staining=yellow brown), and 3 (strong staining=brown). The extent (0–100%) of reactivity was scored as follows: 0 (<5% positive cells), 1 (5–25% positive cells), 2 (25–50% positive cells), 3 (51–75% positive cells), and 4 (>75% positive cells). Scores of 0–2 were classified as low expression, whereas all other scores were classified as high expression.^26,27^

Two pathologists without knowledge of the clinicopathological variables independently scored staining on each slide. Staining assessment and the allocation of tumors by the two pathologists were similar. Cases with discrepancies were simultaneously reviewed by the original two pathologists and a senior pathologist until a consensus was reached.

### Mouse xenograft model

All xenograft experiments were performed in accordance with the guidelines of the Laboratory Animal Ethical Committee at Chengdu Medical College. All experimental protocols were approved by the Laboratory Animal Ethical Committee at Chengdu Medical College. An A549 lung metastasis model was established to investigate the effect of PAX6 on cell migration and invasion in vivo. Female nude mice (6–8 weeks of age, 20–22 g) were purchased from Dossy Experimental Animals Co., Ltd (Chengdu, China). Before performing the experiment, mice were randomly assigned to four treatment groups (siRNA control, si-PAX6, vector control, and PAX6 overexpression). Briefly, 5 × 10^5^ A549 cells were resuspended in 100 µL PBS and injected into the lateral tail veins of mice, which were euthanized 36 days later to count pulmonary metastatic nodules. Lung tissues were fixed in 10% formalin. Representative lung tumors were removed, fixed and embedded in paraffin, and sections of each lung tissue sample were stained routinely with hematoxylin and eosin (H&E).

A subcutaneous A549 model was established to investigate the effect of PAX6 on cisplatin (*cis*-diamminedichloroplatinum, CDDP) cytotoxicity and proliferation in vivo. A549 cells (1 × 10^6^) were suspended in 100 μL of serum-free RPMI-1640 medium and subcutaneously injected into each mouse. Seven days after cell implantation, mice in each group were intraperitoneally injected with 100 μL of PBS or CDDP (4 mg/kg per mouse per week). Tumor growth was measured every 7 days using Vernier calipers. Tumor volume was measured according to the formula V = (a × b^2^)/2, where a and b are the maximal and minimal diameters in millimeters, respectively. At day 28 after cell injection, the mice were killed, and tumors were weighed immediately after dissection.

### RT^2^ profiler PCR array

To investigate the molecular mechanism of PAX6 in NSCLC metastasis, we used the Human Tumor Metastasis RT^2^ Profiler PCR Array, which is designed to represent 84 genes known to be involved in metastasis (KangChen Bio-tech Company, Shanghai, China). A549 cells were divided into four groups (control, si-PAX6, vector control, PAX6 overexpression). Genes selected for this array included several classes of proteins involved in cell adhesion, ECM components, cell cycle, cell growth and proliferation, apoptosis, transcription factors, regulators, and other genes related to tumor metastasis. RNA isolation, DNase treatment, and RNA cleanup were performed according to the manufacturer’s protocol (Qiagen, Hilden, Germany). The isolated RNA was reverse transcribed into cDNA using the RT^2^ First Strand Kit (Invitrogen). PCR was performed using the RT^2^ SYBR Green qPCR Master Mix (Invitrogen) on an ABI PRISM7900 instrument (Applied Bio-systems, Foster City, CA). Data normalization was based on correcting all Ct values for the average Ct values of several housekeeping genes present on array. Each assay was conducted in triplicate.

### Chromatin immunoprecipitation (ChIP) assay

A ChIP assay kit (Beyotime Institute of Biotechnology, Jiangsu, China) was purchased and the assay was conducted following the manufacturer’s instructions. Briefly, A549 cells were fixed by adding formaldehyde to a final concentration of 1%, and the cells were incubated at 37 °C for 10 min to allow cross-linking of endogenous proteins and DNA. Following three washes with cold PBS supplemented with 1 mM PMSF, the cells were resuspended in buffer containing 1% sodium dodecyl sulfate and 1 mM PMSF and lysed by sonication using a sonicator (Scientz-IID, China). After centrifugation, the supernatant was collected and chromatin in the supernatant was immunoprecipitated with anti-PAX6 or normal mouse IgG control antibodies. Input control and DNA obtained from the immunoprecipitation were amplified by PCR using primers specific to the *ZEB2* promoter containing the PAX6 binding site as follows: forward 5′-CACTACTGTTCCGATTTGGT-3′, reverse 5′-AGAAAGCTGACTGGGTGC-3′.

### Statistical analysis

Survival curves were established by the Kaplan–Meier method and compared by the log-rank test. A Chi-square test was used to assess the correlation between PAX6 expression and clinicopathological characteristics. Cox regression models were employed for univariate and multivariate analyses. Each in vitro experiment was performed at least three times independently. Data analysis was performed with the statistical program GraphPad Prism 7.0 (GraphPad, San Diego, CA, USA). Results were presented as the mean ± standard deviation (SD) unless otherwise indicated. Statistical analyses were performed using two-tailed Student’s *t*-tests to analyze the significance of the differences between two groups. *P* *<* 0.05 was considered statistically significant.

## Results

### PAX6 expression is associated with lung cancer patient prognosis

**Fig. 1:**
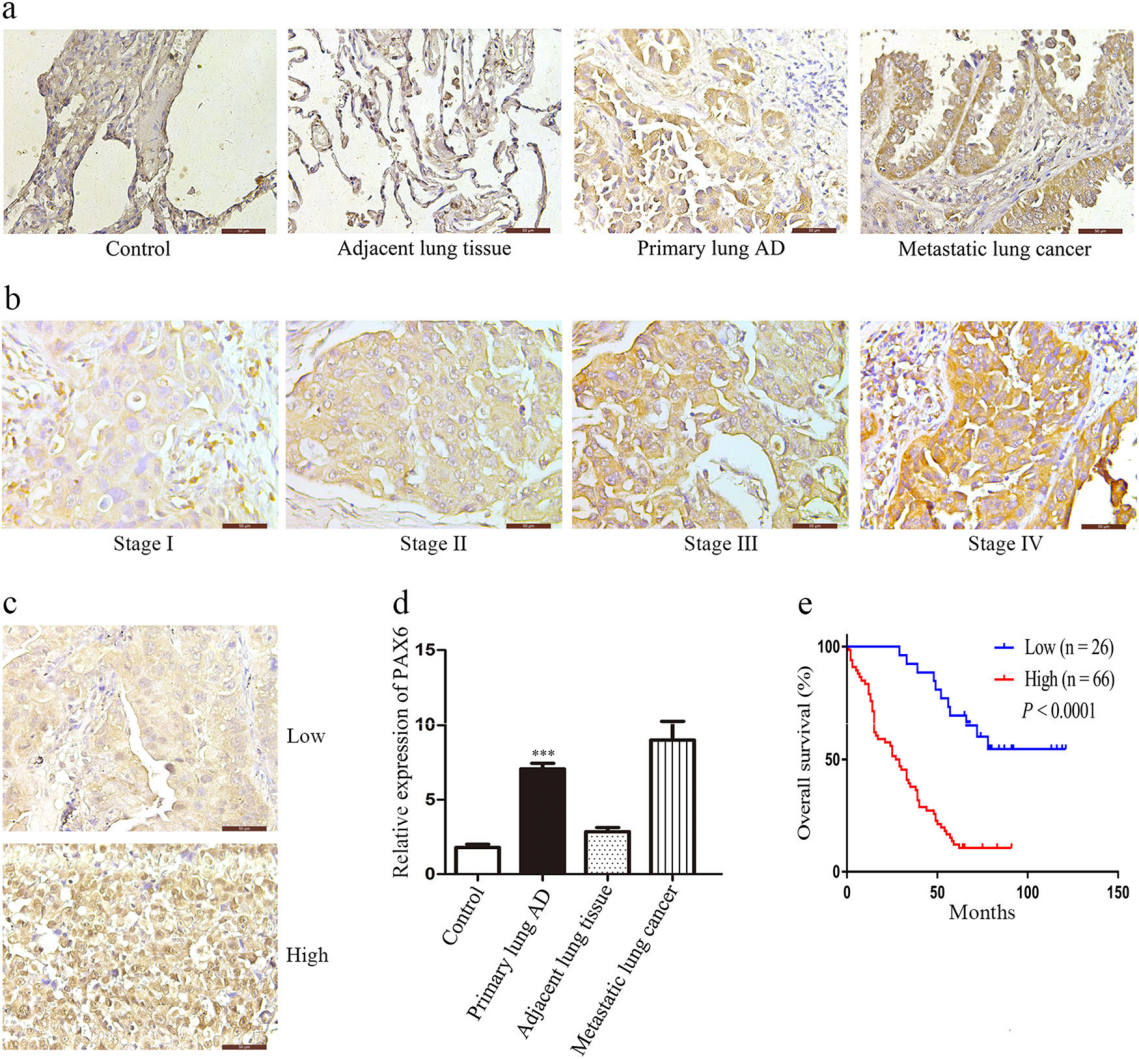
PAX6 expression is correlated with poor prognosis in lung cancer. **a**, **d** Representative images of IHC staining for PAX6 in samples from human NSCLC tissue arrays (magnification, 400×). Semi-quantitative results of PAX6 expression levels in lung cancer tissue arrays. ***P < 0.001, two-tailed Student’s *t*-test. **b**, **c** Representative images of PAX6 IHC staining in samples from a human NSCLC tissue array (magnification, 400×). **e** Kaplan–Meier analysis of overall survival of 92 NSCLC patients. Each subgroup was divided into low- (below or equal to the median value) and high-PAX6 expression groups (above the median value). *P* *<* 0.0001, log-rank test

To investigate the clinical significance of PAX6 expression in patients with NSCLC, we first examined its expression in a human NSCLC tissue array (HLug-Ade050CD-01) by IHC. PAX6 was significantly upregulated in tumor tissues compared to normal lung tissues (Fig. 1a, d). We further investigated whether PAX6 expression is correlated with the clinical outcome of human lung cancer. To this end, we analyzed another human NSCLC tissue array (HLugA180Su05), which contained gene expression profiles of 92 human lung adenocarcinomas with clinical follow-up information. Then, we separated the samples into groups of low or high-PAX6 expression based on IHC staining score (Fig. 1b, c) and we evaluated OS by Kaplan–Meier analysis.

Notably, PAX6 expression was correlated with advanced clinical stage (*P* *=* 0.0006) and regional lymph node spread (*P* *=* 0.0001) based on the Chi-square test (Table 1). In addition, clinical stage, regional lymph node spread, and PAX6 expression were independent predictors of OS based on multivariate Cox regression analysis (Table 2). Importantly, Kaplan–Meier survival analysis showed that lung cancer patients with high-PAX6 expression had shorter OS (Fig. 1e). These results indicated that PAX6 expression is upregulated in human lung cancer tissues, which correlates with poor prognosis in lung cancer; this suggests that this protein might promote cancer cell invasion during malignant progression.

**Table 1:** Correlation between PAX6 expression in tumor tissues and clinicopathologic characteristics of non-small-cell lung cancer patients

Characteristic	Patients	PAX6 expression		*P*-value
		Low (*n* = 26)	High (*n* = 66)	
Gender				0.9437
Male	49	14	35	
Female	43	12	31	
Age (years)				0.6951
<60	36	11	25	
≥60	56	15	41	
Tumor size (cm)				0.2080
<4	40	14	26	
≥4	52	12	40	
Primary tumor (T)				0.1813
T_1_–T_2_	69	22	47	
T_3_–T_4_	23	4	19	
Tumor stage				0.0006^***^
I+II	60	24	36	
III+IV	32	2	30	
Lymph node metastasis				0.0001^***^
No	38	19	19	
Yes	54	7	47	

***P < 0.001.

**Table 2:** Univariate and multivariate analyses of prognostic factors in non-small cell lung cancer patients

Characteristic	Univariate analysis			Multivariate analysis		
	HR	95% CI	*P*-value	HR	95% CI	*P*-value
Gender						
Male vs. Female	0.733	0.457–1.176	0.198			
Age (years)						
<60 vs.≥60	0.951	0.587–1.540	0.837			
Tumor size (cm)						
<4 vs.≥4	1.357	0.839–2.196	0.214			
Primary tumor (T)						
T_1_–T_2_ vs. T_3_–T_4_	1.424	0.838–2.419	0.191			
Tumor stage						
I+II vs. III+IV	2.586	1.583–4.224	0.000***	1.684	1.016–2.790	0.043*
Lymph node metastasis						
No vs. Yes	2.614	1.558–4.388	0.000***	1.592	1.042–2.743	0.038^*^
PAX6 expression						
Low vs. High	5.179	2.661–10.081	0.000***	4.089	1.998–8.368	0.000***

^*^P < 0.05, ***P < 0.001.

### PAX6 promotes migration, invasion, and EMT in NSCLC cells

To determine the effect of PAX6 on cell invasion during NSCLC malignant progression, we first established A549 and SPC-A-1 cell lines that constitutively and stably overexpress exogenous PAX6. Increased expression was confirmed by western blot analysis and upregulated *PAX6* mRNA was determined by RT-qPCR (Additional file 1, Fig. S1a, f). Based on wound healing assays, PAX6-overexpressing cells migrated significantly faster than vector control cells (Additional file 1, Fig. S1b, g). Furthermore, results from transwell assays showed that cell migration (Additional file 1, Fig. S1d, i) and invasion (Additional file 1, Fig. S1e, j) were significantly increased with PAX6 overexpression in A549 and SPC-A-1 cells. Further, western blotting and IF showed that PAX6 overexpression markedly reduced the levels of the epithelial marker E-cadherin, but increased mesenchymal markers N-cadherin, vimentin, and FSP-1 (Additional file 1, Fig. S1c, h), suggesting that PAX6 can promote EMT in NSCLC cells. These data indicated that this protein has a critical role in promoting NSCLC cell migration, invasion, and EMT.

### PAX6 is essential for EMT in NSCLC

**Fig. 2:**
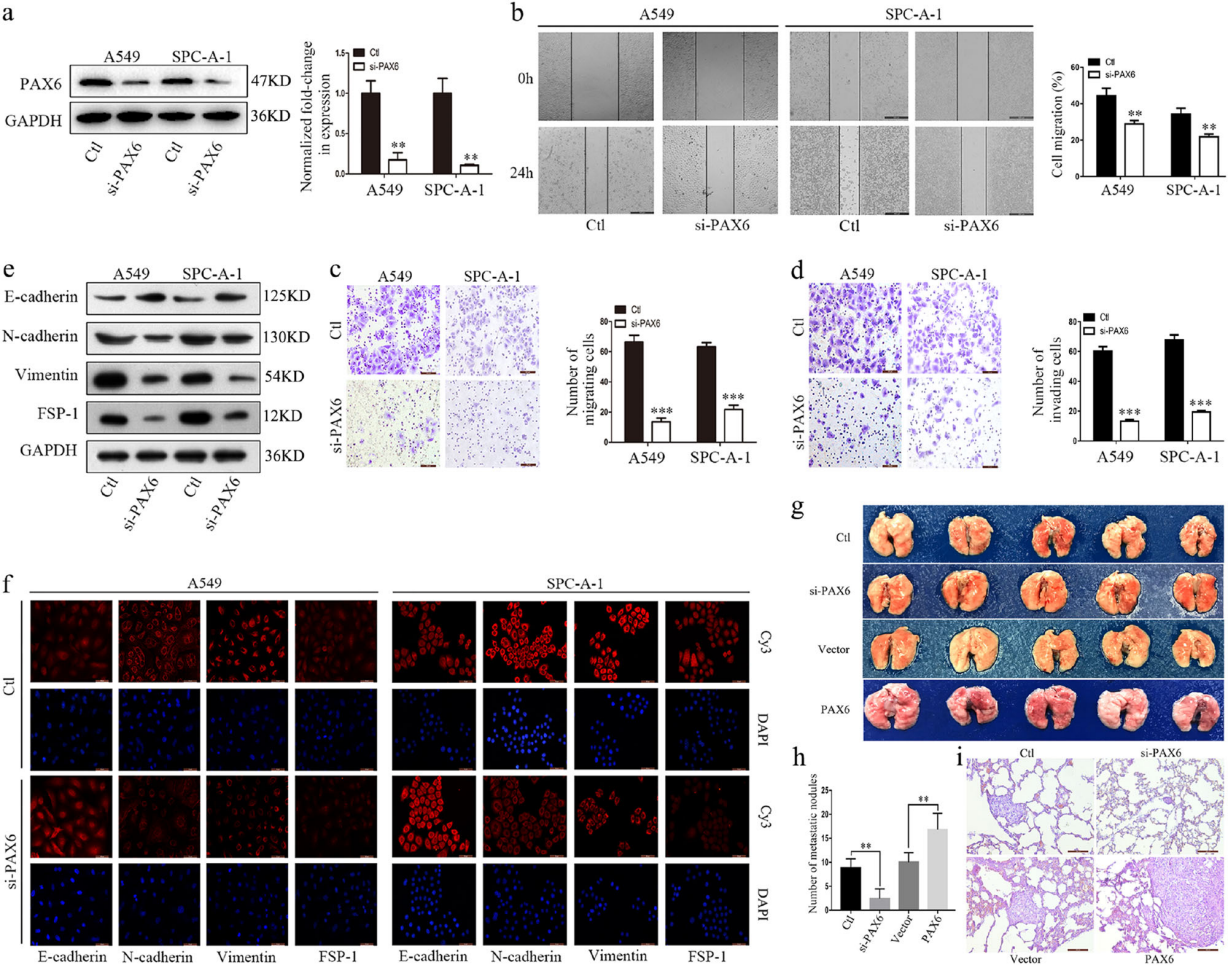
PAX6 is essential for EMT and promotes NSCLC cell metastasis in a xenograft mouse model. **a** Expression of PAX6 in control (Ctl) and PAX6-reduced (si-PAX6) cells was detected by western blot and RT-qPCR assays. **b** Representative images and quantitative analysis of cell migration based on wound-healing assays (scale bar, 500μm). Representative images and quantitative analysis of cell migration **c** and invasion **d** based on Transwell assays (scale bar, 100μm). **e, f** Analysis of EMT markers by western blotting and IF staining (scale bar, 50μm). **g** Cells from four A549 groups (control, si-PAX6, vector, PAX6) were injected into the tail vein of mice, and animals were monitored for 36 d until killed. Lung tissues were then examined for tumor metastasis. **h** Average number of metastatic tumor nodules from indicated groups of mice (*n* = 5 for each group). Results are shown as the mean ± SD from five mice. **i** Representative H&E images of lung tissue sections from each group (scale bar, 100μm). **P < 0.01, ***P < 0.001, two-tailed Student’s *t*-test

Stable PAX6 knockdown cell lines were also generated to explore the function of endogenous PAX6 in NSCLC cell lines. Because si-h-PAX6_002 resulted in the strongest knockdown (Additional file 1, Fig. S2), we utilized this lentivirus-mediated siRNA to induce PAX6 knockdown in NSCLC cell lines. We confirmed that these cells had lower protein and mRNA levels of PAX6 than control cells (Fig. 2a). As assessed by wound healing (Fig. 2b) and transwell assays (Fig. 2c, d), PAX6 knockdown obviously inhibited cell migratory and invasive abilities in A549 and SPC-A-1 cells. Moreover, PAX6 knockdown led to a marked increase in the epithelial marker E-cadherin, whereas the expression of the mesenchymal markers N-cadherin, vimentin, and FSP-1 was decreased (Fig. 2e, f).

In addition, we used the human normal pulmonary epithelial cell line BEAS-2B as a control. BEAS-2B cells were also infected with PAX6 knockdown or overexpression vectors, and the infection efficiency was measured by western blotting and RT-qPCR (Additional file 1, Fig. S3a). Meanwhile, western blotting and immunofluorescence showed that PAX6 knockdown increased the expression of E-cadherin, but decreased the expression of N-cadherin, vimentin, and FSP-1; as expected, PAX6 overexpression had the opposite effects (Additional file 1, Fig. S3b, c).

### PAX6 promotes NSCLC cell metastasis in vivo

To investigate the effect of PAX6 on lung cancer cell metastasis using an *in-vivo* model, we injected mice with A549 cells expressing si-control, si-PAX6, vector-control, or PAX6-overexpressing lentivirus via the tail vein. Although A549/vector- and A549/PAX6-injected animals developed lung tumors, more and larger tumors were found with PAX6 overexpression. In parallel, the A549/si-PAX6-injected animals had fewer and smaller tumors than the A549/si-control-injected animals, suggesting that PAX6 expression is critical for the extravasation and invasion of lung cancer cells (Fig. 2g). The colonization of cancer cells was also significantly affected by PAX6 modulation; the number of tumor nodules in mice injected with A549/si-PAX6 cells was decreased relative to that in mice that received A549/control cells on day 36, whereas opposite effects were observed for the A549/PAX6-injected group (Fig. 2h). The lung tissues were further examined by H&E staining to confirm the presence of tumors (Fig. 2i). Collectively, these data suggest that PAX6 is capable of promoting metastasis in NSCLC.

### PAX6 depresses sensitivity to cisplatin in human NSCLC cells by regulating stem cell transformation

**Fig. 3:**
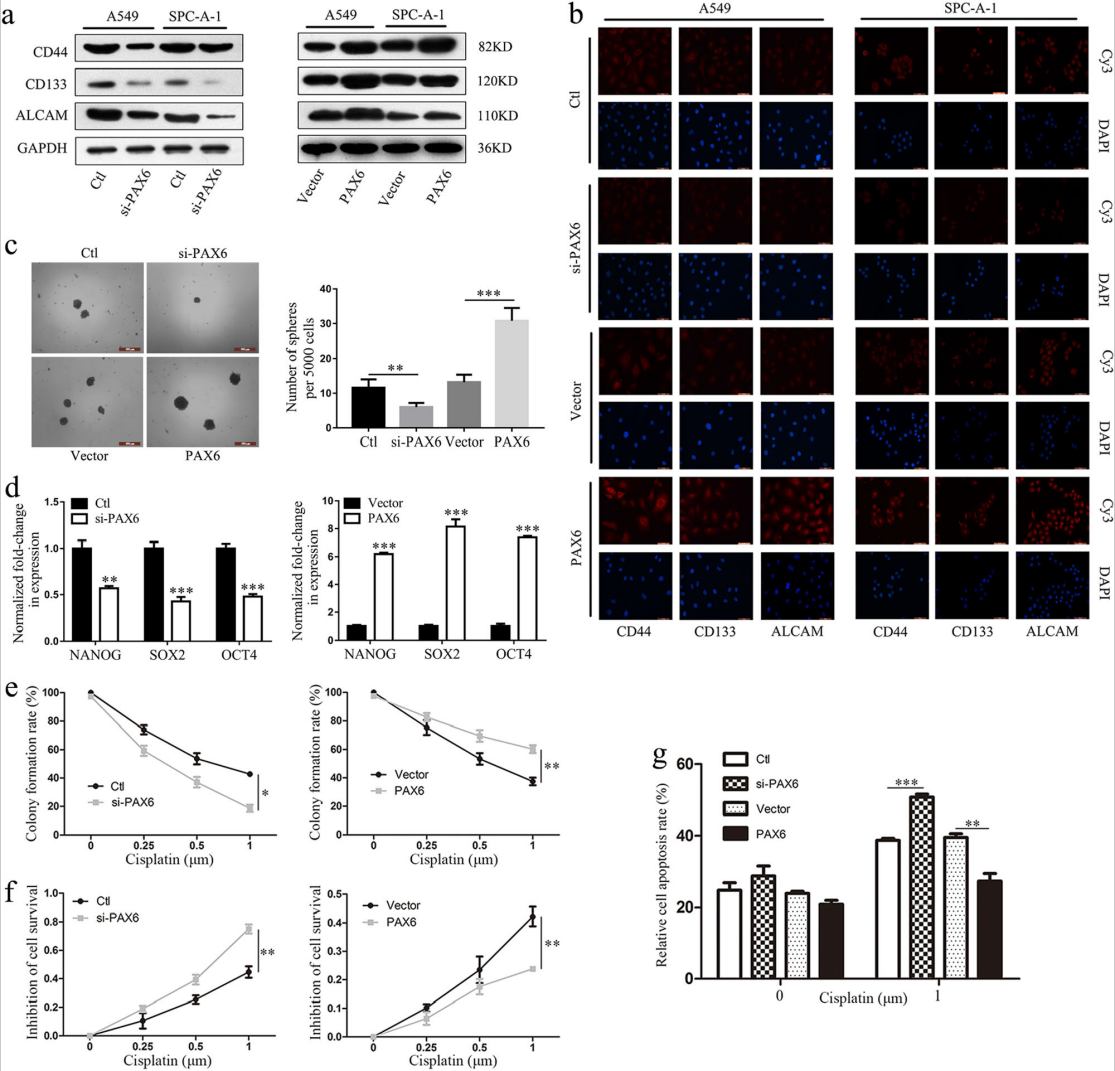
PAX6 depresses sensitivity to cisplatin in human NSCLC cells by regulating stem cell transformation. **a**, **b** Expression of CD44, CD133, and ALCAM in the indicated groups of A549 and SPC-A-1 cells were detected by western blotting and immunofluorescence staining (scale bar, 50μm). **c** Representative images and quantitative analysis of sphere formation in the indicated groups of A549 cells (scale bar, 500μm). **d** mRNA levels of the stemness-associated genes, *NANOG*, *SOX2*, and *OCT4*, by RT-qPCR in the indicated groups of A549 cells. **e** Quantitative analysis of colony formation assays in the four groups of cells treated with four concentrations of cisplatin (0, 0.25, 0.5, 1μm). **f** CCK-8 assays were performed to determine the resistance or sensitivity to cisplatin at four concentrations; **e**, **f**: *P < 0.05, **P < 0.01; two-way ANOVA. **g** Quantitative analysis of flow cytometry results to detect apoptosis in the four groups of cells. **P < 0.01, ***P < 0.001; two-tailed Student’s *t*-test

Because many studies have shown that EMT confers resistance to chemotherapy and cancer stem cells exhibit chemoresistance,^28–30^ we suspected that PAX6 might affect the chemotherapeutic response by regulating stem cell transformation. The relationship between PAX6 expression and the expression of lung cancer stem cell biomarkers including CD44, CD133, and ALCAM^31^ was detected by western blot and immunofluorescence analyses. We found that PAX6 expression significantly correlated with the expression of lung cancer stem cell biomarkers in two human NSCLC cell lines (Fig. 3a, b). In addition, PAX6 knockdown significantly decreased and PAX6 overexpression significantly increased sphere formation in A549 cells (Fig. 3c). Moreover, we found that expression levels in A549 spheres of three stemness-related transcription factors, NANOG, SOX2, OCT4, significantly correlated with PAX6 expression (Fig. 3d). These results suggest that PAX6 can promote cancer stem cell-like traits in NSCLC cancer.

We further explored the functional significance of PAX6 expression with respect to CDDP resistance. Notably, colony formation and CCK-8 assays revealed that PAX6 could inhibit CDDP-induced A549 cell death (Fig. 3e, f; Additional file 1, Fig. S4a), suggesting that this protein enhances cellular resistance to CDDP therapy. Similar findings were observed in apoptosis assays, wherein A549/PAX6 tumor cells exhibited a lower rate of apoptosis and were resistant to CDDP treatment (Fig. 3g; Additional file 1, Fig. S4b).

**Fig. 4:**
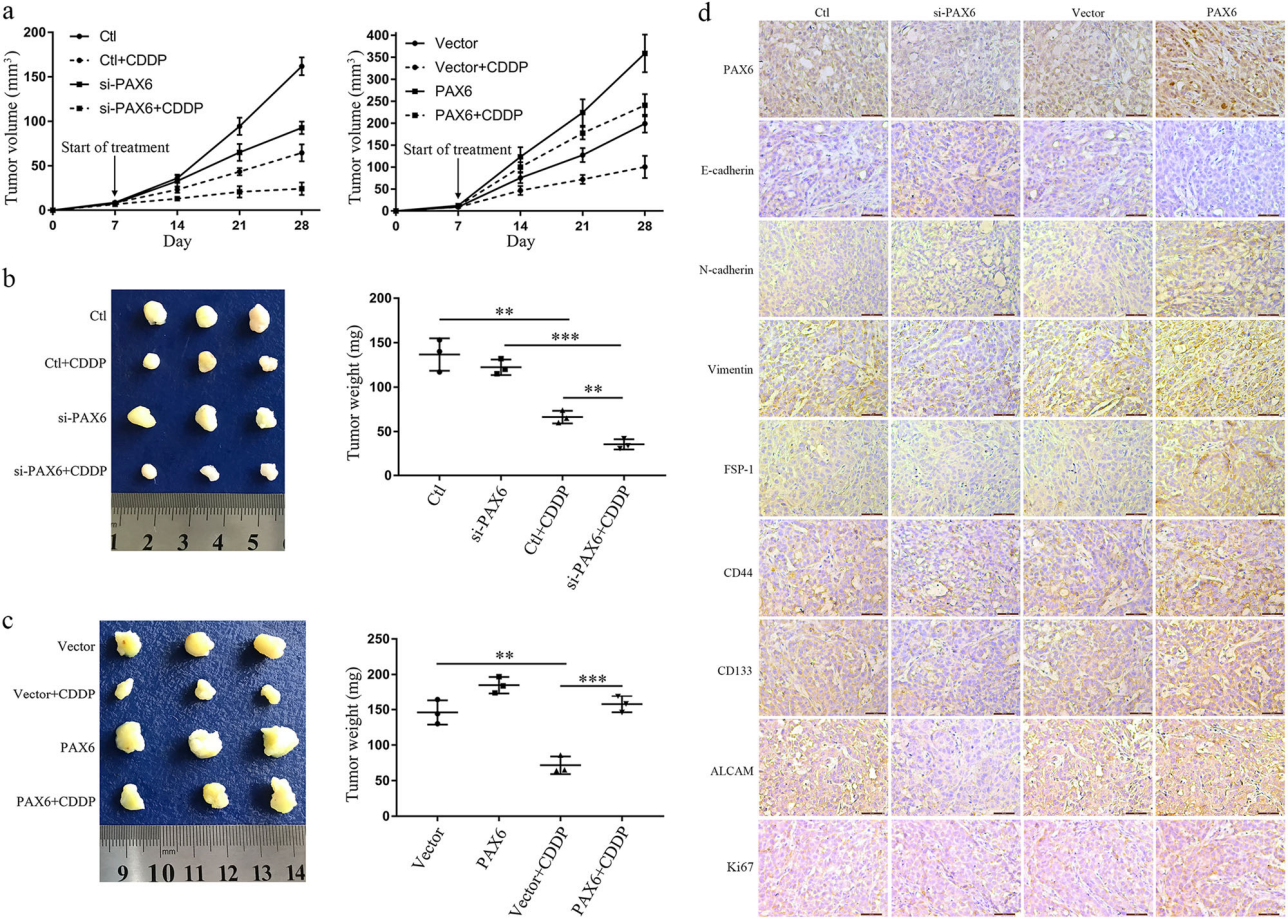
A subcutaneous tumor model for detecting cisplatin sensitivity in nude mice using A549 cells. **a** Tumor volumes of subcutaneous Ctl or si-PAX6 or Vector or PAX6 overexpression xenografts during 21 days of treatment with PBS or CDDP. **b**, **c** Representative tumors isolated from nude mice and average tumor weights in the indicated groups; **P < 0.01, ***P < 0.001; two-tailed Student’s *t*-test. **d** IHC staining of PAX6, EMT-associated genes (E-cadherin, N-cadherin, vimentin, FSP-1), stem cell biomarkers (CD44, CD133, ALCAM), and the proliferation marker Ki67 in subcutaneous tumors of mice injected with Ctl vs. si-PAX6, Vector vs. PAX6 (scale bar, 50μm)

Moreover, we established a subcutaneous tumor model in nude mice using A549 cells. Compared to the control group, PAX6 knockdown significantly restored CDDP efficacy as evidenced by decreases in tumor volume and weight (Fig. 4a). In contrast, PAX6 strongly inhibited the efficacy of CDDP, promoting the growth of xenografts (Fig. 4b, c). In a follow-up experiment, we found that upregulated PAX6 expression elevated the expression of EMT marker genes (N-cadherin, vimentin, FSP-1), stem cell biomarkers (CD44, CD133, ALCAM), and the proliferation marker Ki67, yet it inhibited E-cadherin in tumor tissues as assessed by IHC. Conversely, downregulated PAX6 expression inhibited the expression of these proteins and elevated E-cadherin expression (Fig. 4d).

### ZEB2 is a direct transcriptional regulatory target of PAX6

**Fig. 5:**
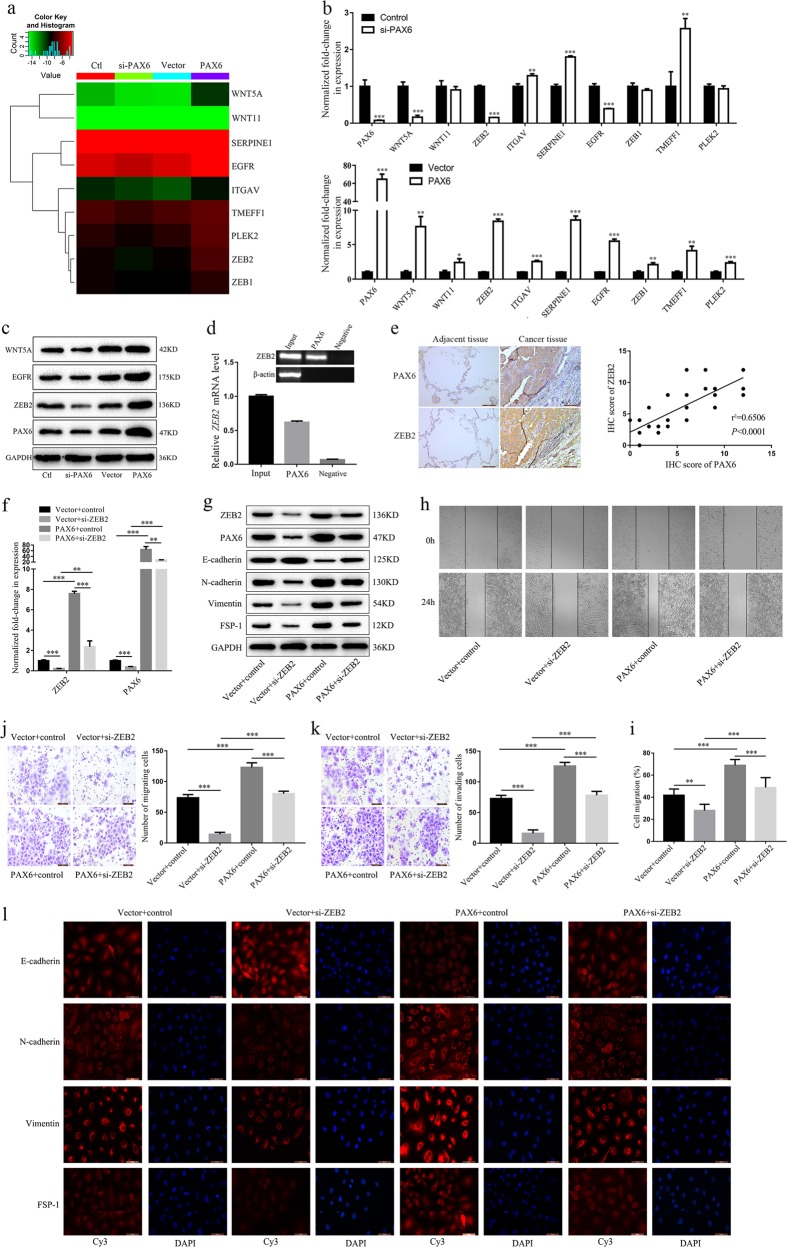
ZEB2 is a direct transcriptional regulatory target of PAX6. **a** Heatmap illustrating the expression of nine representative EMT-related genes in four groups of A549 cells. **b** RT-qPCR analysis of the mRNA expression of nine potentially differentiated genes based on the Human Tumor Metastasis RT^2^ Profiler PCR Array in A549 cells. **c** The expression levels of three representative genes were compared by western blotting. **d** A ChIP assay was performed using an antibody against PAX6 or control IgG in A549 cells. Immunoprecipitated DNA was analyzed by PCR followed by agarose gel electrophoresis. **e** Representative results of IHC staining (scale bar, 100 μm) and the relationship between PAX6 and ZEB2 protein levels in human lung cancer samples. **f** After ZEB2 knockdown (si-ZEB2), mRNA levels of *ZEB2* and *PAX6* were evaluated by RT-qPCR. **g** After ZEB2 knockdown, ZEB2, PAX6 and EMT markers were detected by western blot. **h** Representative images and **i** quantitative analysis of cell migration based on wound-healing assays (scale bar, 500μm). **j**, **k** Representative images (left panels) and quantitative analysis (right panels) of **j** cell migration and **k** invasion by transwell assay (scale bar, 100μm). **l** Analysis of EMT markers by IF staining (scale bar, 50μm). **P < 0.01, ***P < 0.001; two-tailed Student’s *t*-test

To investigate the molecular mechanism through which PAX6 is associated with NSCLC metastasis, we used the Human Tumor Metastasis RT^2^ Profiler PCR Array. After performing this array-based analysis (Additional file 1, Fig. S5a), we selected nine genes that were upregulated in A549/PAX6 cells and downregulated in A549/si-PAX6 cells as compared to the respective control groups (Fig. 5a; Additional file 1, Fig. S5b). To confirm differential expression, mRNA levels were assessed by RT-qPCR (Fig. 5b). We found that only three genes (*WNT5A*, *EGFR*, *ZEB2*) were differentially regulated in response to PAX6 modulation. Western blot and qPCR assays indicated that ZEB2 was the most suitable gene for further study (Fig. 5c).

Sequence analysis of the *ZEB2* promoter revealed a potential core binding sequence (AAATGATGAGTAAA) for PAX6. To determine if PAX6 can specifically bind the *ZEB2* promoter in lung cancer cells, we analyzed binding activity in A549 cells by ChIP assays. The results demonstrated significant PAX6 binding to the *ZEB2* promoter at day 3 (Fig. 5d). To further confirm the correlation between PAX6 and ZEB2, we analyzed PAX6 and ZEB2 protein levels in 15 pairs of NSCLC and adjacent tissues, and we observed a significant positive correlation between PAX6 and ZEB2 (*r*^2 ^= 0.6506, *P* *<* 0.0001; Fig. 5e).

To assess the effects of ZEB2 on PAX6 expression and its potential role in NSCLC, we used three siRNA oligonucleotides to knock down ZEB2 levels in A549 cells and in a stable A549 cell line with high-PAX6 expression. Based on western blotting and RT-qPCR results, we used the cells transfected with si-h-ZEB2_003 (si-03) for subsequent experiments (Additional file 1, Fig. S6a). We found that ZEB2 knockdown significantly decreased levels of PAX6 mRNA and protein in A549 cells (Fig. 5f, g) and reversed the elevation of PAX6 and ZEB2 levels in a PAX6-overexpressing A549 cell line (Fig. 5f, g). Similarly, ZEB2 knockdown inhibited the migration and invasion of A549 cells and PAX6-overexpressing A549 cells in wound healing (Fig. 5h, i) and transwell (Fig. 5j, k) assays, respectively. In addition, ZEB2 knockdown markedly increased the expression of E-cadherin and decreased the expression of N-cadherin, vimentin, and FSP-1, which suggests that ZEB2 may inhibit the EMT in A549 cells (Fig. 5g, l). As expected, the effect of PAX6 overexpression on levels of proteins involved in the EMT was reversed by ZEB2 knockdown (Fig. 5g, l). Taken together, our results revealed that ZEB2 is a direct transcriptional regulatory target of PAX6, and the PAX6-ZEB2 axis may play an important role in NSCLC.

### The PAX6-ZEB2 axis regulates EMT via the PI3K/AKT signaling pathway

**Fig. 6:**
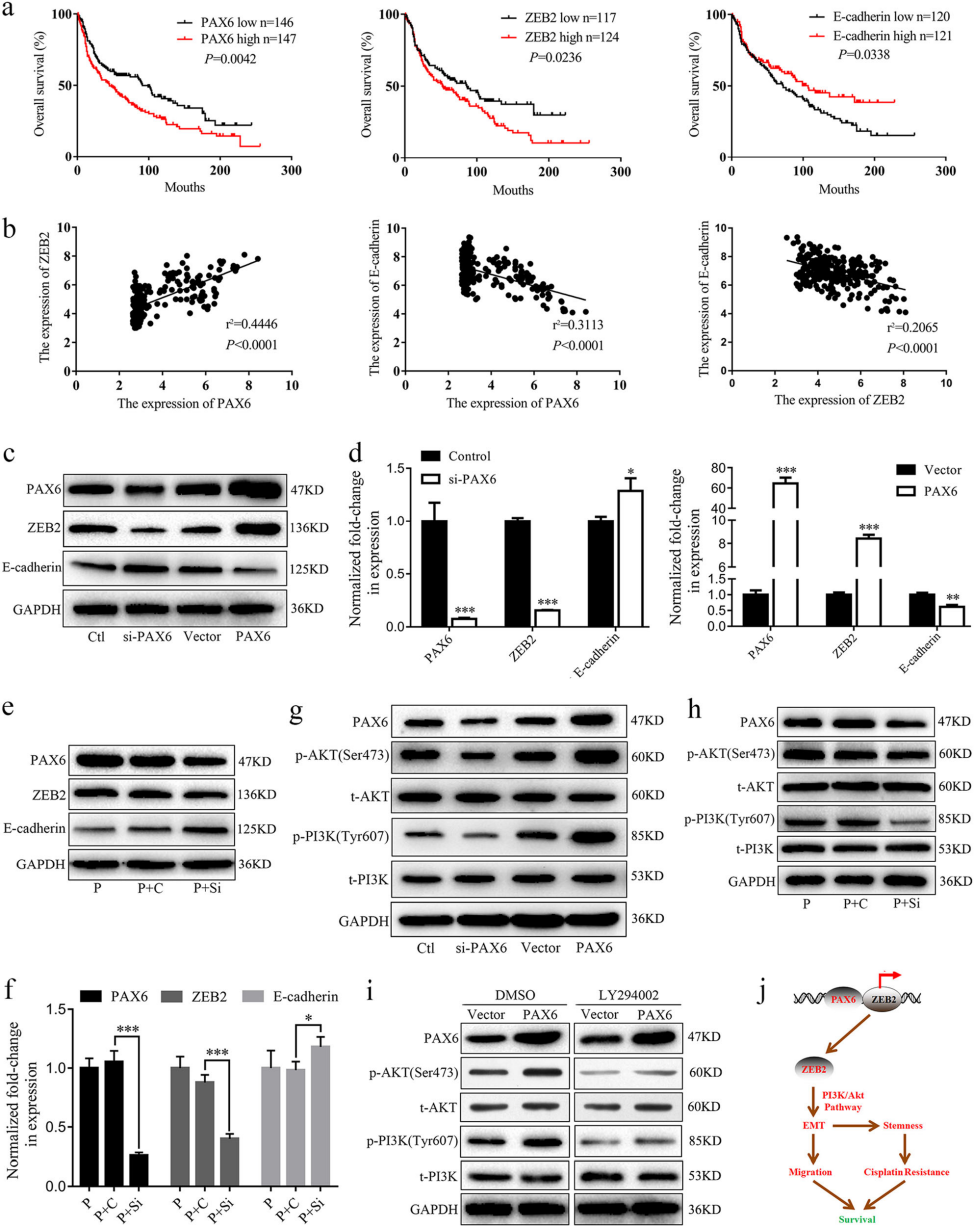
The PAX6-ZEB2 axis mediates EMT via PI3K/AKT signaling in NSCLC. **a**, **b** The GEO dataset GSE30219 was downloaded for survival and correlation analyses. **c**, **d** Expression of PAX6, ZEB2, and E-cadherin in the indicated groups of A549 cells was detected by western blot and RT-qPCR assays. **e**, **f** Western blot analysis of PAX6, ZEB2 and E-cadherin was performed in A549/PAX6 (P), A549/PAX6-control (P + C), or A549/PAX6-silenced (P+Si) cells. The mRNA levels of *PAX6, ZEB2* and E-cadherin were analyzed by RT-qPCR in the indicated groups. **d**, **f**: *P < 0.05, **P < 0.01, ***P < 0.001, two-tailed Student’s *t*-test. **g**, **h** Western blot analysis of PAX6, and phospho- and total AKT and PI3K for the indicated groups. **i** A549/Vector and A549/PAX6 were incubated with medium containing DMSO or LY294002 (25 mM) for 24 h prior to harvesting and lysing. Western blot analysis of AKT and PI3K (phosphorylated and total). **j** Model depicting the role of the PAX6-ZEB2 axis in NSCLC. PAX6 directly binds the promoter region of *ZEB2*. Further, the PAX6-ZEB2 axis regulates EMT via the PI3K/AKT signaling pathway, thereby mediating cell migration, stem cell transformation, and cisplatin resistance, ultimately affecting survival in NSCLC patients

Previous studies have reported that ZEB2 strongly correlates with induction of invasion^32–34^ and mediates E-cadherin downregulation via the PI3K/AKT signaling pathway.^35–37^ Network analysis using STRING database yielded ZEB2 as the core molecule interacting with 10 other genes, including E-cadherin/CDH1 (Additional file 1, Fig. S6). In addition, we performed survival analyses in a lung cancer GEO dataset (GSE30219). Low expression of E-cadherin and high expression of PAX6 and ZEB2 were associated with poor prognosis in lung cancer (Fig. 6a). PAX6 expression positively correlated with ZEB2 expression, but negatively correlated with E-cadherin expression. Further, we found a negative correlation between ZEB2 and E-cadherin expression (Fig. 6b).

We also investigated the effects of PAX6 on ZEB2 and E-cadherin by RT-qPCR and western blotting. Our data clearly indicated that ZEB2 could be downregulated by PAX6 knockdown and upregulated by PAX6 overexpression, whereas opposite results were found for E-cadherin expression (Fig. 6c, d). Moreover, PAX6-mediated ZEB2 upregulation at the protein and mRNA levels was reversed by PAX6 knockdown, whereas E-cadherin downregulation was reversed by PAX6 knockdown (Fig. 6e, f).

The levels of phosphorylated PI3K (p-PI3K) and phosphorylated AKT (p-AKT) in A549/PAX6 cells were dramatically increased compared to those in control cells, whereas opposite effects were observed in A549/si-PAX6 cells (Fig. 6g). The increased levels of p-PI3K and p-AKT observed after PAX6 overexpression were reduced through downregulation of PAX6 in A549 cells (Fig. 6h). In addition, p-PI3K and p-AKT were significantly enhanced by PAX6, which was reversed by the addition of the PI3K-AKT inhibitor, LY294002 (25 μM, 24 h; Fig. 6i). However, total PI3K (t-PI3K) and total AKT (t-AKT) levels were not changed, indicating that PAX6 can activate PI3K/AKT signaling in lung cancer.

## Discussion

Based on GLOBOCAN estimates, ~18.1 million new cancer cases and 9.6 million cancer deaths occurred in 2018, worldwide. Lung cancer is the leading cause of cancer-related death among males and has surpassed breast cancer as the leading cause of cancer-associated death among females.^38^ Despite advances in treatment, metastasis remains a life-threatening feature of cancer and is primarily responsible for cancer patient mortality.^3^

Previous studies have shown that the downregulation of PAX6 inhibits the proliferation and invasion of NSCLC cells.^39^ In addition, PAX6 expression is associated with poor prognosis in lung cancer.^14,23^ However, a previous study reported conflicting results, suggesting that PAX6 is a positive prognostic marker in NSCLC in node-positive patients.^40^ These differences could be attributed to a lack of sufficient data and clarity regarding the underlying mechanism of the role of PAX6 in this disease. The current study showed that higher PAX6 expression significantly correlated with reduced duration of OS in lung cancer. Further, we found a significant positive correlation of PAX6 expression with both clinical stage and regional lymph node metastasis. To better study the role of PAX6 in NSCLC, we conducted functional and mechanistic experiments.

EMT plays a significant role in tumor invasion and metastasis.^9,41^ In agreement herewith, we found that PAX6 markedly downregulated the levels of E-cadherin, but increased N-cadherin, vimentin, and FSP-1 expression, whereas opposite results were observed after PAX6 knockdown. These results demonstrated that PAX6 can promote lung cancer cell (A549 and SPC-A-1) migration and invasion in vitro. To investigate the effect of PAX6 on lung cancer metastasis in vivo, we established an A549 model of lung metastasis. The results suggested that PAX6 can promote the metastasis of NSCLC cells. Chemoresistance is usually accompanied by metastasis, which inhibits the efficiency of current cancer therapies.^42^ Our results confirm the role of PAX6 in both tumor cells and animals in stimulating NSCLC resistance to cisplatin treatment by regulating stem cell transformation.

Notably, PAX6 is an important transcription factor in humans; however, the molecular mechanism through which PAX6 regulates lung cancer metastasis remains unclear. To detect targets of PAX6, we used the Human Tumor Metastasis RT^2^ Profiler PCR Array, which contains 84 primer pairs that amplify genes involved in human metastasis. We found that PAX6 could directly bind the *ZEB2* promoter region, identifying ZEB2 as a direct transcriptional target of PAX6. ZEB2 acts as a transcriptional repressor and strongly correlates with the induction of invasion.^34^ ZEB2 was also identified in a large-scale screen for cancer-related genes, which demonstrated its putative role in oncogenic transformation.^35,36^ An inverse correlation between ZEB2 and E-cadherin expression levels was observed in several epithelial tumor cell lines.^37^

Western blotting and RT-qPCR assays further supported that ZEB2 could be downregulated by PAX6 knockdown and upregulated by PAX6 overexpression, whereas opposite results were found for E-cadherin expression. The regulation of E-cadherin by ZEB2 has been shown to depend on the PI3K/AKT signaling pathway.^36^ Here, we found that overexpression of PAX6 increases the levels of p-AKT and p-PI3K, which were reduced by PAX6 downregulation. p-PI3K and p-AKT were significantly enhanced by PAX6, which was reversed by the addition of the PI3K-AKT inhibitor, LY294002. Therefore, these data suggest that PAX6 can mediate E-cadherin downregulation through the PI3K/AKT signaling pathway by directly binding the promoter region of *ZEB2* in NSCLC cells.

In conclusion, this study reveals a novel link between the binding of PAX6 to the promoter region of *ZEB2* and lung cancer progression. The PAX6-ZEB2 axis promotes metastasis by mediating E-cadherin downregulation through the PI3K/AKT signaling pathway, thereby mediating cell migration, stem cell transformation, and cisplatin resistance, ultimately affecting survival in NSCLC patients (Fig. 6j). Our results demonstrate that PAX6 could be a potential therapeutic target for NSCLC.

## Supplementary information

Table S1, Fig. S1, Fig. S2, Fig. S3, Fig. S4, Fig. S5, Fig. S6
