# Supplementary material for: The PAX6-ZEB2 axis promotes metastasis and cisplatin resistance in non-small cell lung cancer through PI3K/AKT signaling
Source: Cell Death Dis. 2019 Apr 25;10(5):349. doi: 10.1038/s41419-019-1591-4 (PMC6483988; doi:10.1038/s41419-019-1591-4)
Supplement: Supplementary file 1 — Table S1, Fig. S1, Fig. S2, Fig. S3, Fig. S4, Fig. S5, Fig. S6 [file 41419_2019_1591_MOESM1_ESM.doc]

**Additional file 1**

**Table S1. Sequences of the primers used in this study**

| **Target gene** | **Forward primer sequence** | **Reverse primer sequence** |
| --- | --- | --- |
| **PAX6** | CAGAACAGTCACAGCGGAGT | CCGTTGGACACCTGCAGAAT |
| **E-cadherin** | ATGAAGAAGGAGGCGGAGAAGAGG | TGCAACGTCGTTACGAGTCACTTC |
| **ZEB2** | TGACCTGCCACCTGGAACTCC | ACTTGATGTGCTCCTTCAGTGATGTC |
| **WNT5A** | GCTGGCAGACTTCCGCAAGG | GAAGCGGCTGTTGACCTGTACC |
| **WNT11** | TGAAGGACTCGGAACTCGTCTATCTG | TGTCTTGTTGCACTGCCTGTCTTG |
| **ITGAV** | CAGGAGTTCCAAGAGCAGCAAGG | CATCAGAGCCACGATCCATGAAGAG |
| **PLEK2** | AGCCTGAGCACTGTGGAGTT | GCTGCTGGCCTGAATGT AAT |
| **SERPINE1** | AGCAGCAGATTCAAGCAGCTATGG | GCGTCTGTGGTGCTGATCTCATC |
| **EGFR** | GCGTTCGGCACGGTGTATAAGG | ACGGTGGAGGTGAGGCAGATG |
| **ZEB1** | CCAAGAACAGGACTCAAGACATCTCAG | TTGCCGTATCTGTGGTCGTGTG |
| **TMEFF1** | GCTACACGTCGGTGCTTCTG | CAGTTGATGCTCTTGCCTTTGC |
| **NANOG** | AAGGTCCCGGTCAAGAAACAG | CTTCTGCGTCACACCATTGC |
| **OCT4** | CTGGGTTGATCCTCGGACCT | CCATCGGAGTTGCTCTCCA |
| **SOX2** | TGGACAGTTACGCGCACAT | CGAGTAGGACATGCTGTAGGT |
| **β-actin** | CCTGGCACCCAGCACAAT | GGGCCGGACTCGTCATAC |


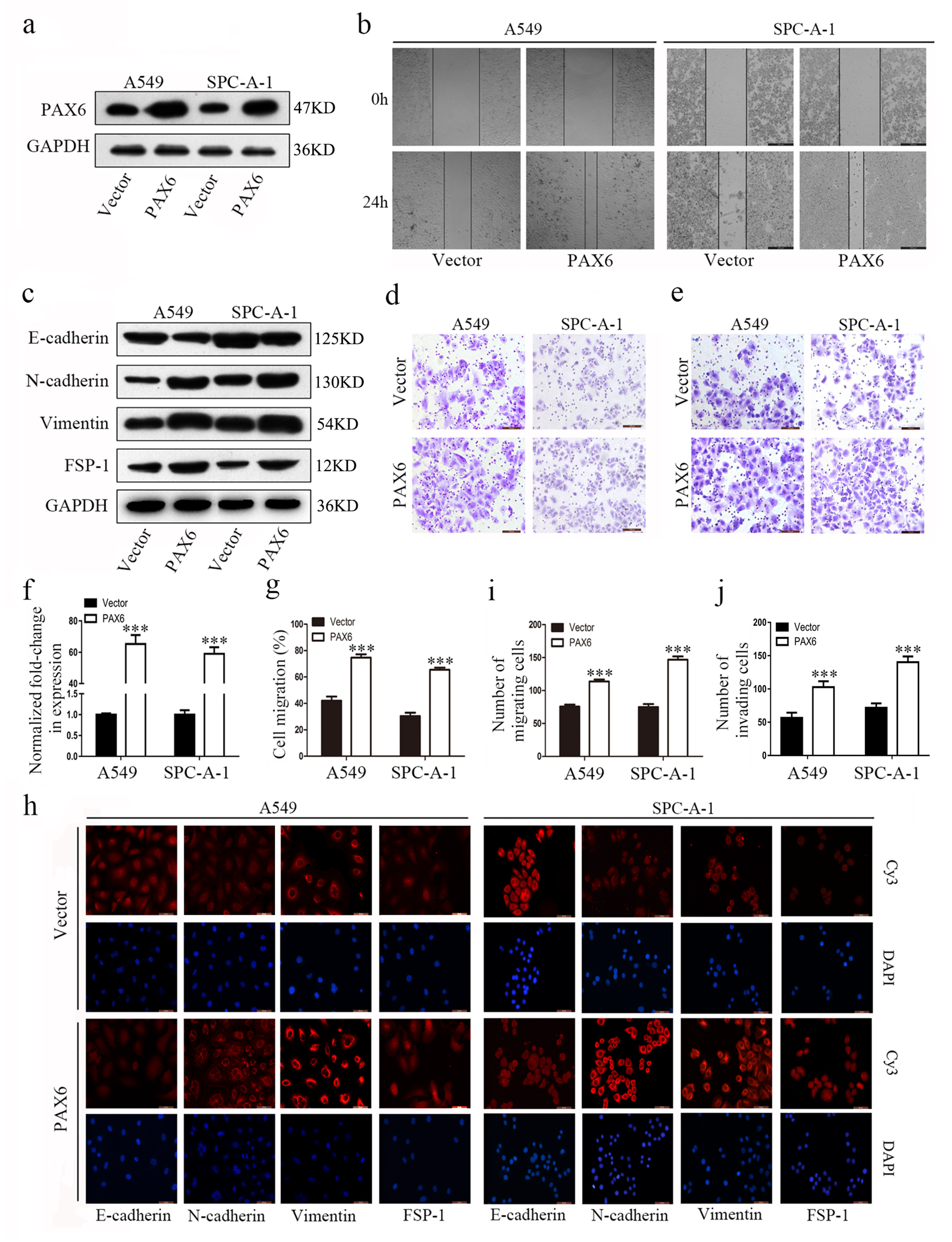
**Fig. S1.** PAX6 promotes NSCLC cell migration, invasion, and EMT *in vitro*. (**a, f**) Expression of PAX6 in empty vector control (Vector) and PAX6-overexpressing (PAX6) cells was detected by western blot and RT-qPCR assays. GAPDH served as a loading control. (**b, g**) Representative images and quantitative analysis of cell migration based on wound-healing assays (scale bar, 500 μm). (**c, h**) Analysis of EMT markers by western blotting and IF staining (scale bar, 50 μm). Representative images and quantitative analysis of cell migration (**d, i**) and invasion (**e, j**) based on Transwell assays (scale bar, 100 μm). Histograms represent the mean ± s.d. based on threeindependent experiments. ****P* < 0.001, two-tailed Student’s *t*-test.


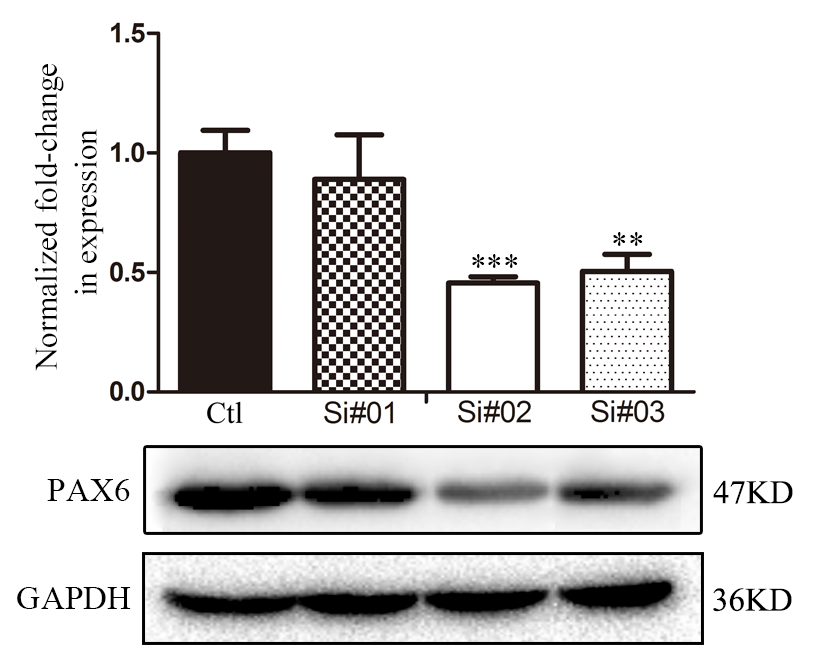
**Fig. S2.** Screening of siRNA sequences for PAX6 knockdown. The efficiency of three si-h-PAX6 oligonucleotides was assessed by RT-qPCR and western blot assays in A549 cells. ***P* < 0.01, ****P* < 0.001, two-tailed Student’s *t*-test.


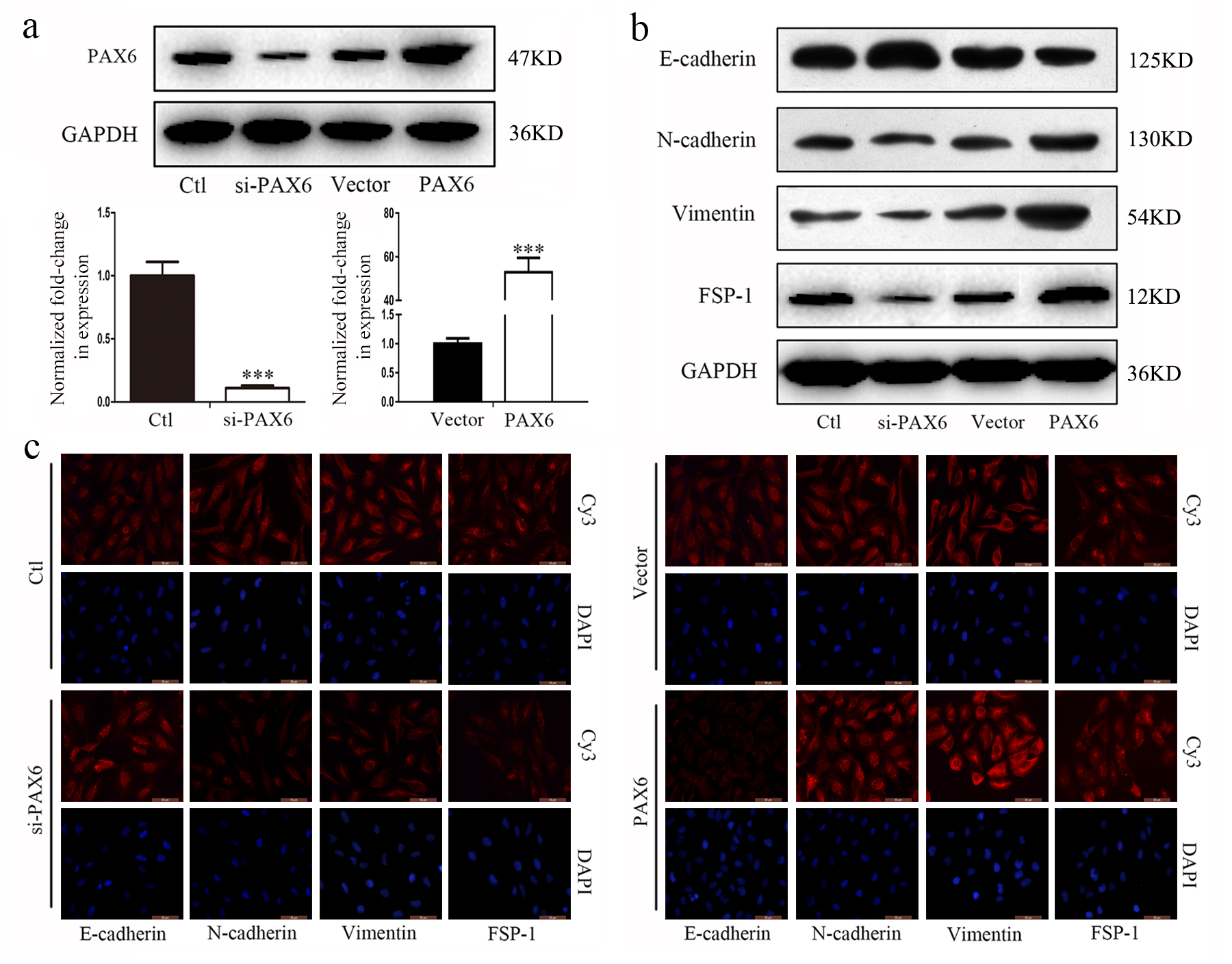
**Fig. S3.** Effect of PAX6 on epithelial-to-mesenchymal transition (EMT) in BEAS-2B human normal pulmonary epithelial cells. (**a**) The expression of PAX6 was detected in Ctl, si-PAX6, Vector, PAX6 cells by western blot and qRT-PCR assays. (**b, c**) Analysis of EMT markers by western blotting and immunofluorescence staining (scale bar, 50 μm). ****P* < 0.001, two-tailed Student’s t-test.


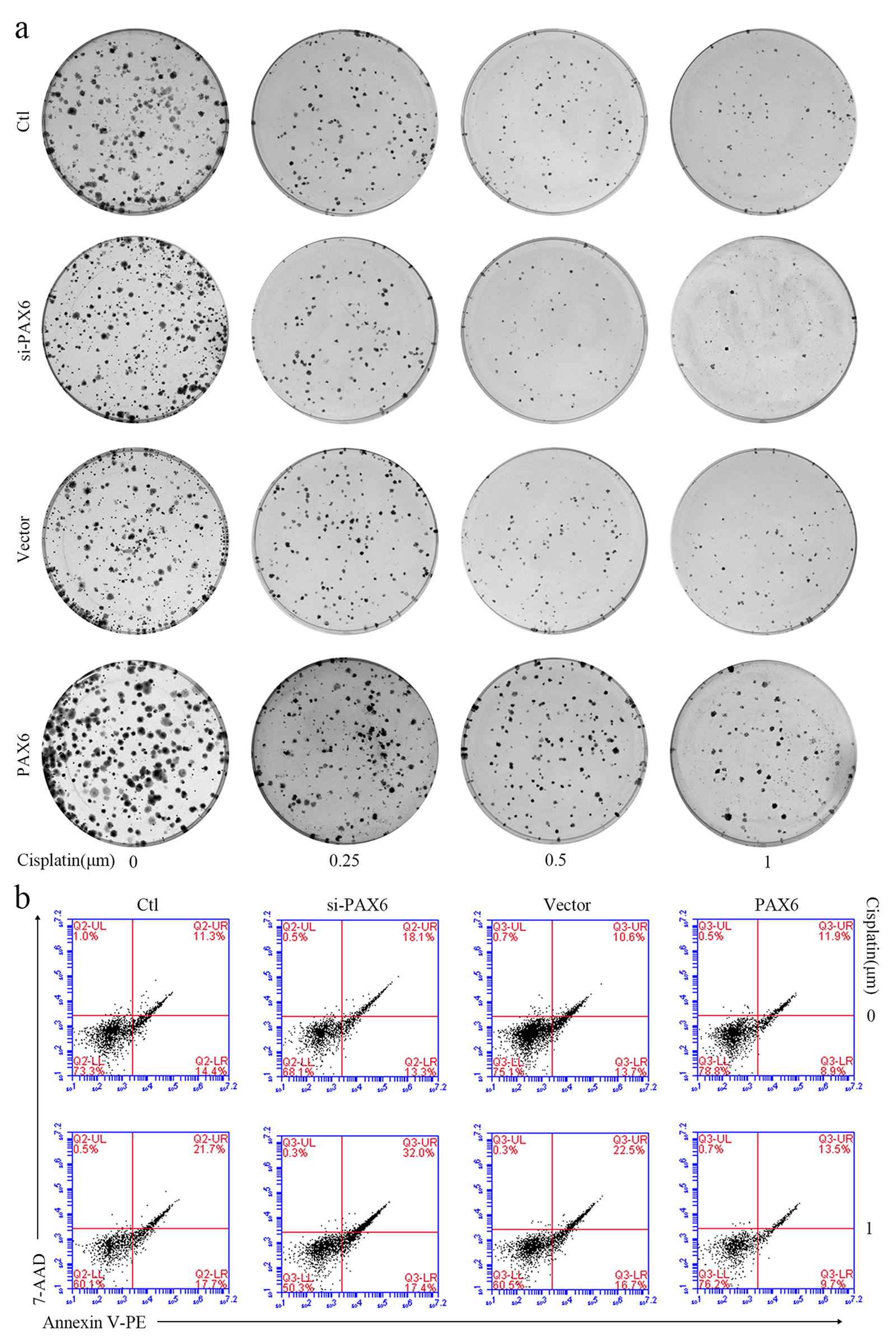
**Fig. S4.** Representative images of colony formation assays and flow cytometry to assess apoptosis. (**a**) Representative images of colony formation assays for the four groups of cells treated with four concentrations of cisplatin (CDDP; 0, 0.25, 0.5, 1 μM). (**b**) Representative flow cytometry images for the four groups of cells treated with CDDP (0, 1 μM).


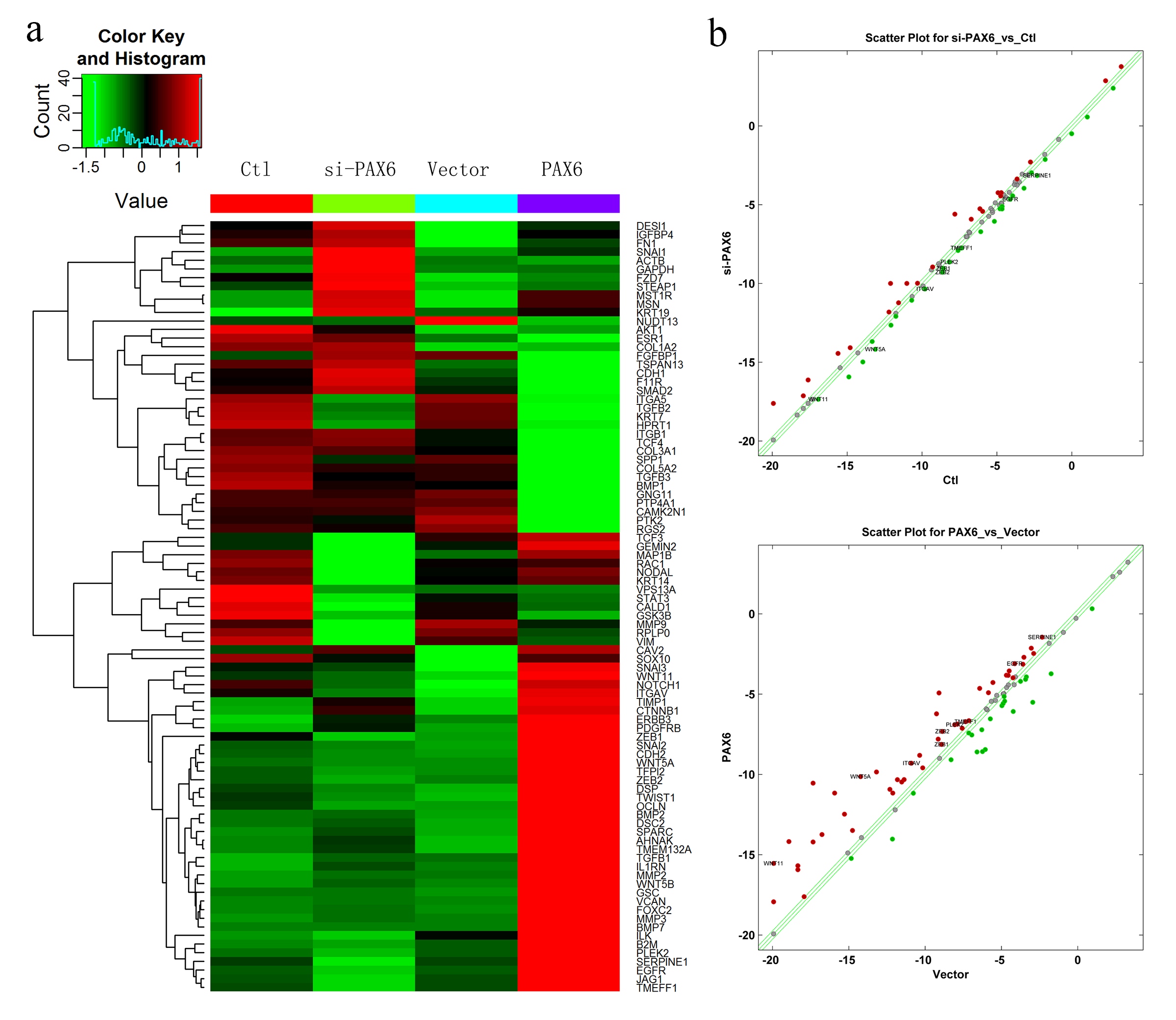
**Fig. S5.** Results of the Human Tumor Metastasis RT^2^ Profiler PCR Array analysis. (**a**) Heatmap illustrating the expression of 84 genes known to be involved in metastasis in Ctl, si-PAX6, Vector, and PAX6 A549 cells. (**b**) Scatter plots revealed that nine genes were upregulated in A549/PAX6 cells and downregulated in A549/si-PAX6 cells as compared to the respective control groups.


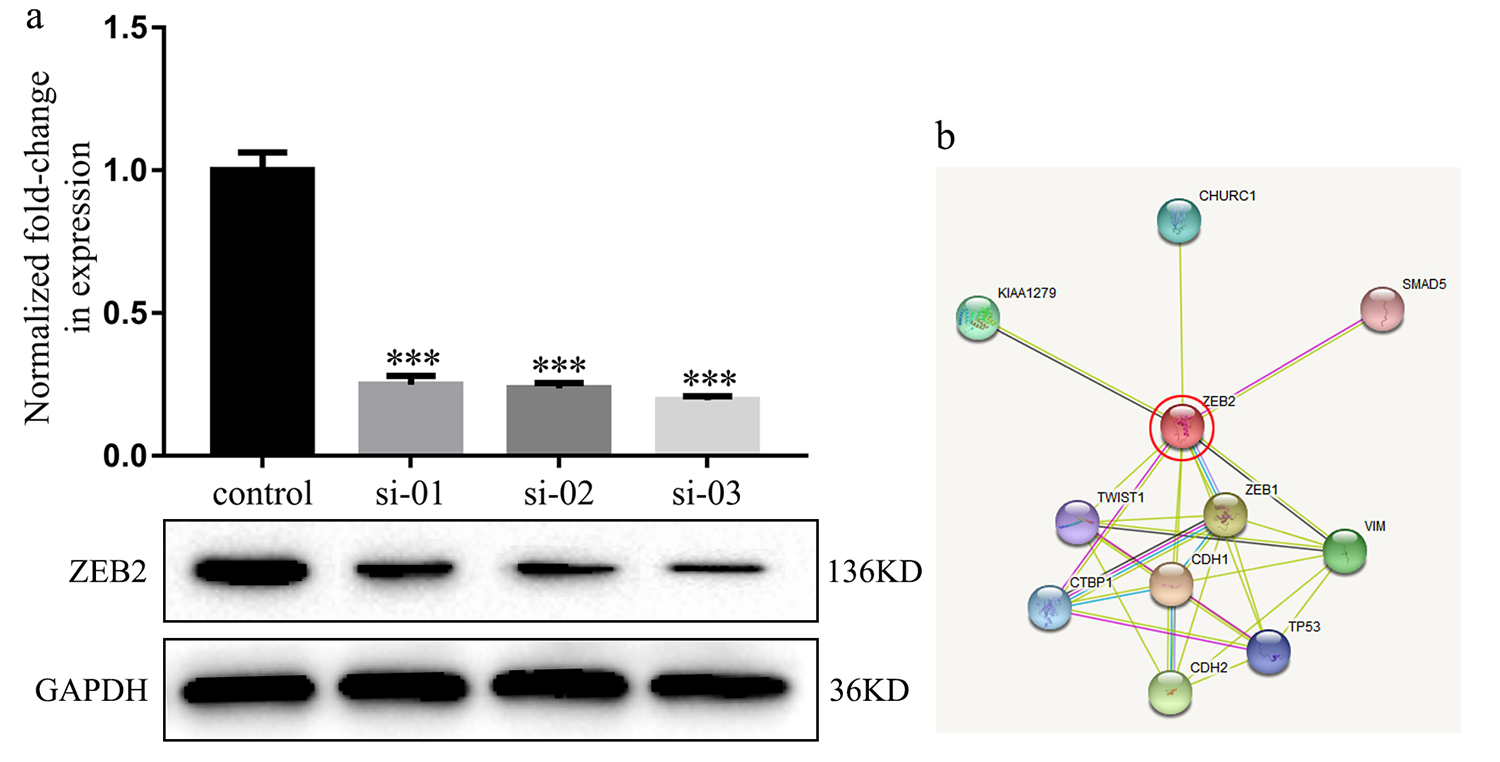
**Fig. S6.** Screening of siRNA sequences for ZEB2 knockdown and the network analysis of ZEB2. (**a**) The efficiency of three si-h-ZEB2 oligonucleotides was assessed by RT-qPCR and western blot assays in A549 cells. ****P* < 0.001, two-tailed Student’s *t*-test. (**b**) Network analysis using STRING database yielded ZEB2 as the core molecule interacting with 10 other genes.
